# Adverse drug events in the prevention and treatment of COVID-19: A data mining study on the FDA adverse event reporting system

**DOI:** 10.3389/fphar.2022.954359

**Published:** 2022-11-24

**Authors:** Qiang Guo, Shaojun Duan, Yaxi Liu, Yinxia Yuan

**Affiliations:** ^1^ Department of Pharmacy, Jincheng People’s Hospital, Jincheng, China; ^2^ Department of Information Technology, Jincheng People’s Hospital, Jincheng, China

**Keywords:** COVID-19, SARS-CoV-2, data mining, adverse drug events, FAERS

## Abstract

**Background:** In the emergent situation of COVID-19, off-label therapies and newly developed vaccines may bring the patients more adverse drug event (ADE) risks. Data mining based on spontaneous reporting systems (SRSs) is a promising and efficient way to detect potential ADEs to help health professionals and patients get rid of the risk.

**Objective:** This pharmacovigilance study aimed to investigate the ADEs of some attractive drugs (i.e., “hot drugs” in this study) in COVID-19 prevention and treatment based on the data from the US Food and Drug Administration (FDA) adverse event reporting system (FAERS).

**Methods:** The FAERS ADE reports associated with COVID-19 from the 2nd quarter of 2020 to the 2nd quarter of 2022 were retrieved with hot drugs and frequent ADEs were recognized. A combination of support, lower bound of 95% confidence interval (CI) of the proportional reporting ratio (PRR) was applied to detect significant hot drug and ADE signals by the Python programming language on the Jupyter notebook.

**Results:** A total of 66,879 COVID-19 associated cases were retrieved with 22 hot drugs and 1,109 frequent ADEs on the “preferred term” (PT) level. The algorithm finally produced 992 significant ADE signals on the PT level among which unexpected signals such as “hypofibrinogenemia” of tocilizumab and “disease recurrence” of nirmatrelvir\ritonavir stood out. A picture of signals on the “system organ class” (SOC) level was also provided for a comprehensive understanding of these ADEs.

**Conclusion:** Data mining is a promising and efficient way to assist pharmacovigilance work, and the result of this study could help timely recognize ADEs in the prevention and treatment of COVID-19.

## 1 Introduction

Since the outbreak of the COVID-19 pandemic around the end of 2019, the world has seen a huge number of infected and death cases (over 497 million confirmed and 6 million death cases when this article was written) (World Health Organization, 2022). This is quite a serious infection disease caused by a newly discovered coronavirus (CoV) whose name was given as “severe acute respiratory syndrome coronavirus 2” (SARS-CoV-2) on 11 February 2020 by the International Committee on Taxonomy of Viruses (ICTV) (World Health Organization, 2021b). CoVs are a group of RNA viruses belonging to the Coronaviridae family discovered in 1960s. Before the COVID-19 pandemic, we have seen two outbreaks of CoV epidemics: SARS-CoV in 2003 and MERS-CoV in 2012 (Umakanthan et al., 2020). In general, most people infected with SARS-CoV-2 may experience mild-to-moderate symptoms including fever, fatigue, cough, and other respiratory illness, and could recover without special treatment. However, for some elderly people and those with underlying health problems such as cardiovascular disease, diabetes, respiratory disease, and cancer, the risk of developing serious situations becomes higher (World Health Organization, 2021a).

In this emergent situation, many unapproved therapies (e.g., antiviral chemical drugs, monoclonal antibodies, and convalescent plasma transfusion) and newly developed vaccines have been tried in the treatment and prevention of this deadly virus disease, which, apart from the efficacy, might raise an unpredictable ADE risk to the patients. In order to help timely identify these ADEs, we performed this pharmacovigilance job.

Pharmacovigilance, also known as drug safety surveillance, plays an important role in ADE research. According to the definition of the World Health Organization (WHO), pharmacovigilance is defined as the science and activities relating to the detection, assessment, understanding, and prevention of drug-related problems. It usually contains two stages: pre-marketing surveillance (data collected from pre-clinical and phase I–III clinical studies) and post-marketing surveillance (data collected after the approval and throughout the market life of a drug). For the former one, there are obviously some inevitable shortcomings such as relatively small sample data, strict enrollment criteria, and large time and money consumption. Post-marketing surveillance, on the other hand, given the rapid development of computer sciences and data mining technologies, may become an even more important and efficient way for pharmacovigilance in the real world (Ibrahim et al., 2016).

There are some prominent spontaneous reporting systems (SRSs) designated for data collection of post-marketing surveillance since 1960s, such as the FAERS of the US Food and Drug Administration and the VigiBase of the World Health Organization (WHO) (US Food and Drug Administration, 2021; World Health Orgarnization, 2021). Annually, these systems receive large numbers of ADE reports and could offer abundant resources for pharmacovigilance research, and in this article, we adopted the reports associated with COVID-19 from the FAERS to perform data mining on the associations between one drug and one ADE.

## 2 Materials and methods

### 2.1 Data source

The FAERS is a publicly available computerized relational database for spontaneous reporting of adverse events and medication errors held by the US Food and Drug Administration (FDA) for monitoring the post-marketing safety of drugs and therapeutic biological products. The data structure complies with the international safety reporting guidance issued by the International Conference on Harmonization (ICH), and the adverse events and therapy indications are all coded on the “preferred term” (PT) level of the Medical Dictionary for Regulatory Activities (MedDRA). This database is a descendant of the former adverse event reporting system (also known as Legacy AERS, which was decommissioned in 2012). The FDA issues FAERS data packages to the public quarterly and provides two formats (ASCII/XML) which could be downloaded from its website (US Food and Drug Administration, 2021). In this study, we used the ASCII format, and reports submitted between the 2nd quarter of 2020 and 2nd quarter of 2022 were retrieved.

In each ASCII format data package, there are seven datasets: patient demographic and administrative information (“DEMO”), drug/biologic information (“DRUG”), adverse events (“REAC”), outcomes for the event (“OUTC”), report sources (“RPSR”), drug therapy start dates and end dates (“THER”), and diagnoses (“INDI”). We imported the DEMO, DRUG, REAC, and INDI datasets into a SQL server to create a local database for this study.

### 2.2 Data preprocessing

The FAERS database is a case/version system in which a new case will be given a “CASEID” (e.g., “17462593”) and a “CASEVERSION” (1 for the first report), and if any follow-up reports of this case are available afterward, new CASEVERSIONs will also be given in a sequentially incremented way (e.g., 2, 3, and 4). According to the FDA’s recommendations for adopting the most recent case version for deduplication, we wrote a program to extract only the most updated reports (i.e., having the max CASEVERSION for a certain case) in which the most complete data were included.

The attribute “PRIMARYID” is a concatenated key of a CASEID and a CASEVERSION which uniquely identifies an FAERS report, and through this key, we could link the four imported datasets together. As our aim was to explore the ADE signals in COVID-19 prevention and treatment, we extracted all reports whose diagnoses in the INDI set matched up with the regular expression “%COVID-19%” or “%SARS-CoV-2%” to form a “COVID-19 case table.” In order to get rid of possible confusions between a cause and a bystander, in the DRUG set, only drugs labeled as “primary suspect” or “secondary suspect” were included.

A program was written to calculate the frequency of drugs and ADEs in this COVID-19 case table. When the frequency was over 200 for a drug or over 20 for an ADE, the drug or the ADE would be marked as frequent.

Since the FAERS permits arbitrary registrations of drugs and this would surely lead to dilutions of some important ADE signals, these frequent drugs we got were all transformed into their generic names in the COVID-19 case table. In addition, ADEs such as “off-label use,” “COVID-19,” “product use in unapproved indication,” and the like were excluded. Then we selected several “hot drugs” (i.e., currently often used or attractive drugs, especially ever recommended by the WHO, in the prevention or treatment of COVID-19) from the frequent drugs to form a list of hot drug and ADE candidates with all the frequent ADEs.

Meanwhile, all the drugs and ADEs of each report in the COVID-19 case table were combined into a transaction to form a transaction set, *T*.

After this step, the preparation of the data we needed for further analysis was completed.

### 2.3 Association rule mining

There are different measures to quantify the reporting proportionality of a signal, such as information component (IC), reporting odds ratio −1.96 standard errors (SE), proportional reporting ratio −1.96 SE, Yule’s Q −1.96 SE, the Poisson probability, and the Chi-square test. It is assumed that when four or more cases are reported for a certain “drug and ADE signal”, these methods are broadly comparable (van Puijenbroek et al., 2002).

In this study, we adopted a combination of support, lower bound of 95% confidence interval (CI) of the proportional reporting ratio (PRR) for the recognition of interesting signals.

Support is the frequency of transactions in the *T* set containing a certain drug and ADE. The proportional reporting ratio (PRR) is the risk ratio of a certain ADE between exposed and comparison groups proposed by S.J.W. Evans et al. (2001) for ADE analyses and has been adopted by regularity agencies (e.g., Eudravigilance—EMEA) in the daily routine pharmacovigilance work (Evans et al., 2001; Ibrahim et al., 2016). To facilitate the discussion of PRR, a 2 × 2 contingency table was created, as shown in Table 1. Here, “Drug_i_” and “ADE_j_,” respectively, refer to a specific drug and ADE, and “!Drug_i_” and “!ADE_j_” represent those other than Drug_i_ and ADE_j_. So PRR could be calculated as follows:

$$PRR=\frac{a/\left(a+b\right)}{c/\left(c+d\right)}.$$

**TABLE 1:** 2 × 2 contingency table for computation of PRR.

	Count of ADE_j_	Count of !ADE_j_	Sum
Drug_i_	a	b	a + b
!Drug_i_	c	d	c + d
Sum	a + c	b + d	a + b + c + d

Here, “a,” in fact, is the frequency of transactions containing both Drug_i_ and ADE_j_, and “c” is the frequency of transactions containing ADE_j_ without Drug_i_. The 95% confidence interval (CI) of the natural logarithm of PRR is calculated as follows:

$$95\%\;CI=PRR\pm e^{1.96\sqrt{\left(\frac{1}{a}-\frac{1}{a+b}+\frac{1}{c}-\frac{1}{c+d}\right)}}.$$

Let the transaction set *T* = {t_1_, t_2_, ... t_m_} be an itemset. We wrote a program to scan *T* to calculate the frequencies of transactions in *T* that contain each specific hot drug, ADE, and the both so as to get “a + b,” “a + c,” and “a” mentioned in Table 1. As “a + b + c + d” equals to the total number of transactions of *T*, all the parameters “a,” “b,” “c,” and “d” we needed were ready.

### 2.4 Screening for significant “hot drug and ADE” signals

According to the research studies conducted before, in this article, a significant signal was recognized when its support (i.e., a) ≥ 4, the lower bound of 95% CI of PRR ≥2.00 (Evans et al., 2001; van Puijenbroek et al., 2002; Ibrahim et al., 2016). Referring to the side effect data of Drugs.com (2021) and UpToDate (2021), these significant ADE signals were, respectively, labeled as unexpected (i.e., ADEs not listed in the aforementioned two databases) and expected.

According to the MedDRA, we also made a combination converting these significant ADEs (including the expected and unexpected ones) on the PT level to the system organ class (SOC) level and drew a heatmap to show the number of reported cases for each signal on the SOC level for a more macroscopic view.

### 2.5 Experiment environment

MySQL (version 5.6.32.0) was used to create a local database from the FAERS quarterly ASCII packages, while Navicat was used for MySQL (version 11.1.13) as a graphic user interface (GUI) tool to process database operations. The proposed data preprocessing, mining algorithm, and graphs were implemented by the Python (version 3.8.0) programming language on the Jupyter Notebook (version 6.3.0). ADEs coded in the preferred term (PT) were transformed into “system organ class” (SOC) through MedDRA (version 24.0). We stored the COVID-19 case table, significant signals, and the other results in Microsoft Office Excel 2017 files.

## 3 Results

**FIGURE 1:**
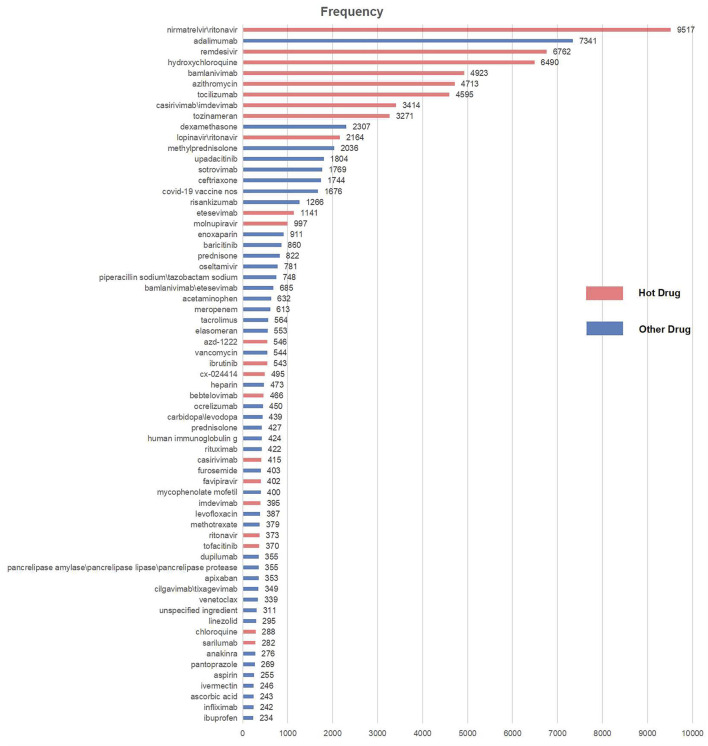
Frequencies of frequent drugs in the COVID-19 table.

**FIGURE 2:**
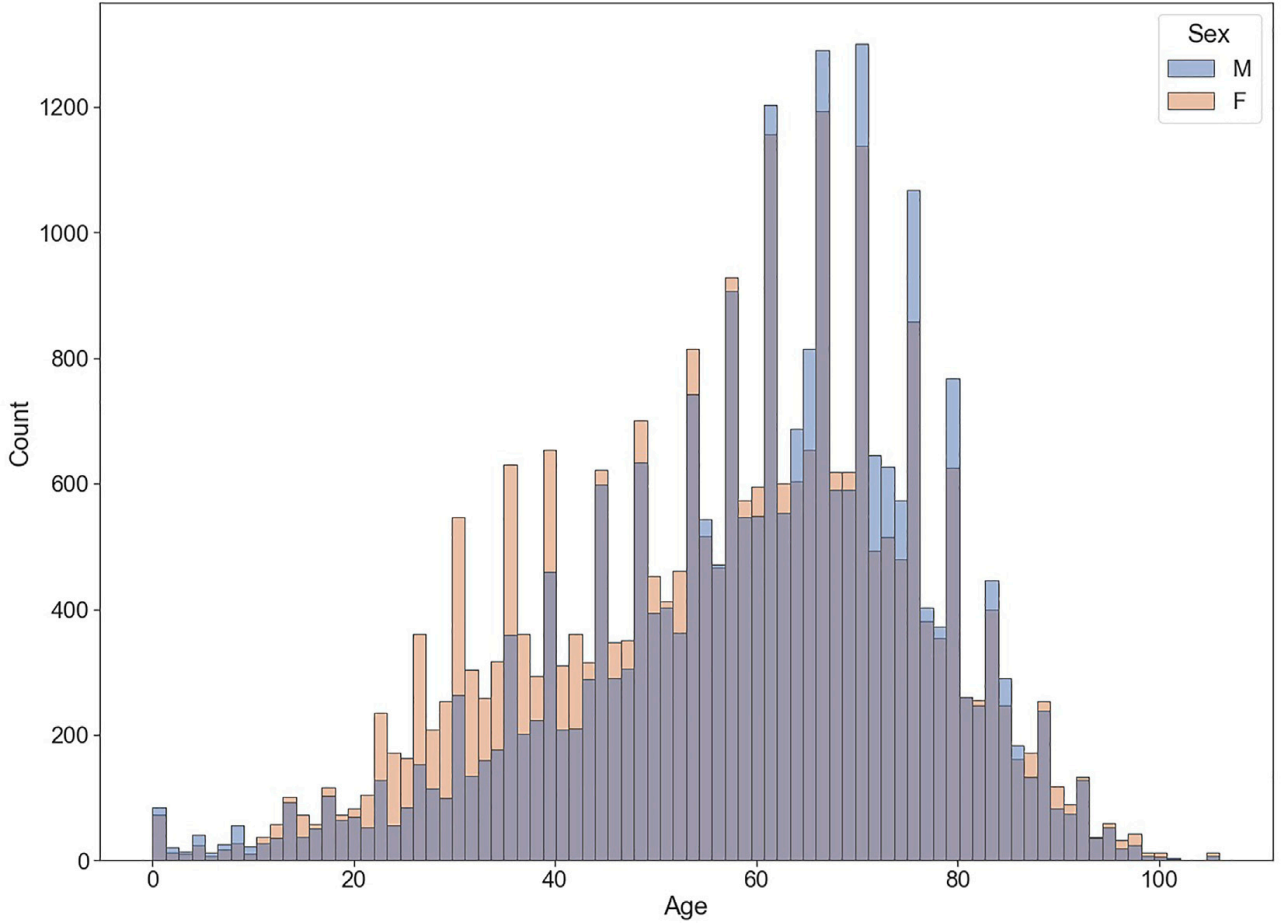
Age–sex distribution of COVID-19 associated cases.

From 2020Q2 to 2022Q2, the FAERS received 70,987 reports whose diagnosis in INDI set matched up with the regular expression %COVID-19% or %SARS-CoV-2%. Table 2 shows some example reports in the COVID-19 case table. After deduplication, the number was reduced to 66,879, with 65 frequent drugs (frequency distributions are shown in Figure 1) and 1,109 frequent ADEs recognized. The age of the patients is 58.41 ± 18.09 (mean ± SD) years, and the proportions of males and females are generally the same (42.70% vs. 48.71%, those without age or sex data excluded). Most reports are from the United States (60.06%). The occupations of reporters are as follows: consumers (24,333, 36.38%), other health professionals (12,546, 18.76%), physicians (10,934, 16.35%), pharmacists (10,817, 16.17%), unknown (8,246, 12.33%), and lawyers (3 cases, <0.01%). The demographic data of COVID-19 cases are shown in Table 3 and Figure 2.

**TABLE 2:** Some reports in the COVID-19 case table.

Primaryid	Caseid	Caseversion	Age	Age_cod	Age_range	Sex	Occr_country	Indication	Drugs	ADEs
1126087917	11260879	17	41	YR	40–49	F	BR	COVID-19	Ascorbic acid, AZD-1222, azithromycin, belimumab, formoterol fumarate, nitazoxanide, and vitamin D NOS	Back pain, blister, circumstance or information capable of leading to medication error, dyspnea, fibrin D dimer increased, headache, hypersensitivity, hypertension, hypoesthesia, inappropriate schedule of product administration, inflammation, off-label use, pain, product dose omission issue, productive cough, protein urine present, pyrexia, skin mass, surgery, swelling face, systemic lupus erythematosus, therapeutic response unexpected, therapy interrupted, urinary tract infection, and vocal cord thickening
1297914822	12979148				22	F	BR	COVID-19 prophylaxis	Azathioprine, belimumab, and prednisone	Circumstance or information capable of leading to medication error, dental operation, inappropriate schedule of product administration, influenza, nasopharyngitis, orthodontic procedure, pneumonia, product dose omission issue, product supply issue, rhinorrhea, social problem, swelling, underdose, urinary tract infection, varicose vein, weight decreased, and weight increased
1355709210	13557092	10	86	YR	80–89	M	US	COVID-19 immunization	Carbidopa \levodopa, pimavanserin tartrate, and prednisone	Abnormal behavior, aspiration, asthenia, bronchitis, cough, decreased appetite, dehydration, delusion, drug ineffective, dysphagia, general physical health deterioration, hallucination, infection, medical device site infection, mental impairment, mental status changes, peripheral swelling, prescribed underdose, sepsis, unresponsive to stimuli, and urinary tract infection
146661614	14666161	4	58	YR	50–59	F	US	US COVID-19 prophylaxis	COVID-19 vaccine NOS and dimethyl fumarate	Abdominal discomfort, flushing, influenza, memory impairment, nasopharyngitis, nausea, vaccination complication, and vomiting

**TABLE 3:** Demographic data of COVID-19 cases.

Items	Cases	
2020Q2	2,404	
2020Q3	4,328	
2020Q4	4,967	
2021Q1	6,600	
2021Q2	7,331	
2021Q3	8,283	
2021Q4	8,811	
2022Q1	11,357	
2022Q2	16,906	
Duplicated	4,108	
Total	66,879	
Sex	Cases	Percentage
Male	28,559	42.70
Female	32,574	48.71
Unknown	5,744	8.59
Age-group (years)		
0–9	441	0.66
10–19	981	1.47
20–29	2,364	3.53
30–39	4,812	7.20
40–49	6,183	9.25
50–59	9,094	13.60
60–69	12,461	18.63
70–79	10,085	15.08
80–89	4,257	6.37
90–99	964	1.44
≥100	44	0.07
Unknown	15,193	22.72
Top 3 reported countries		
US	40,165	60.06
France	2,934	4.39
Italy	2,824	4.22
Reporter occupations		
Consumers	24,333	36.38
Other health professionals	12,546	18.76
Physicians	10,934	16.35
Pharmacists	10,817	16.17
Lawyers	3	<0.01
Unknown	8,246	12.33

Among the 65 frequent drugs, 22 hot drugs were selected to form 22 × 1,109 = 24,398 “Drug and ADE” candidates. After scanning the transaction set *T* with these candidates, PRRs and their 95% CIs of these candidates were calculated. Our mining algorithm finally produced 992 significant signals associated with 22 hot drugs and 603 ADEs on the PT level (the top 3 signals of each hot drug are shown in Table 4, and more details could be found in the Supplementary Appendix).

**TABLE 4:** Top ADE signals of hot drugs on the PT level.^a^

Category	Drug	ADE	a	a + b	c	c + d	95% CI of PRR
Chemical drugs	Azithromycin	Ventricular arrhythmia	16	4,696	7	62,183	30.27 ± 2.43
	Azithromycin	Cardiovascular insufficiency	15	4,696	9	62,183	22.07 ± 2.28
	Azithromycin	Long QT syndrome	75	4,696	52	62,183	19.10 ± 1.42
	Chloroquine	Cardiovascular insufficiency	13	288	11	66,591	273.26 ± 2.21
	Chloroquine	Embolism	13	288	35	66,591	85.88 ± 1.87
	Chloroquine	Brain oedema	13	288	39	66,591	77.07 ± 1.85
	Favipiravir	Inappropriate antidiuretic hormone secretion	8	402	12	66,477	110.24 ± 2.43
	Favipiravir	Neuroleptic malignant syndrome	8	402	16	66,477	82.68 ± 2.32
	Favipiravir	Lymphocyte count increased	4	402	24	66,477	27.56 ± 2.87
	Hydroxychloroquine	Acute generalized exanthematous pustulosis	70	6,489	14	60,390	46.53 ± 1.77
	Hydroxychloroquine	Torsade de pointes	76	6,489	21	60,390	33.68 ± 1.62
	Hydroxychloroquine	Methemoglobinemia	27	6,489	8	60,390	31.41 ± 2.20
	Ibrutinib	Chronic lymphocytic leukemia	10	543	11	66,336	111.06 ± 2.35
	Ibrutinib	White blood cell count abnormal	11	543	22	66,336	61.08 ± 2.05
	Ibrutinib	Onychoclasis	7	543	15	66,336	57.01 ± 2.44
	Lopinavir\ritonavir	Hypertriglyceridemia	44	2,164	22	64,715	59.81 ± 1.66
	Lopinavir\ritonavir	Eosinophilia	73	2,164	44	64,715	49.62 ± 1.45
	Lopinavir\ritonavir	Hyperbilirubinemia	43	2,164	30	64,715	42.86 ± 1.59
	Molnupiravir	Pneumonia aspiration	22	997	70	65,882	20.77 ± 1.61
	Molnupiravir	Drug eruption	13	997	42	65,882	20.45 ± 1.86
	Molnupiravir	Feces soft	5	997	22	65,882	15.02 ± 2.63
	Nirmatrelvir\ritonavir	Disease recurrence	3,463	9,517	69	57,362	302.50 ± 1.27
	Nirmatrelvir\ritonavir	Symptom recurrence	227	9,517	10	57,362	136.82 ± 1.88
	Nirmatrelvir\ritonavir	Dysgeusia	1713	9,517	120	57,362	86.04 ± 1.20
	Remdesivir	Creatinine renal clearance decreased	31	6,762	2	60,117	137.80 ± 4.17
	Remdesivir	Liver function test increased	396	6,762	118	60,117	29.84 ± 1.23
	Remdesivir	Ischemic hepatitis	23	6,762	7	60,117	29.21 ± 2.33
	Ritonavir	Acute generalized exanthematous pustulosis	29	373	55	66,506	94.01 ± 1.55
	Ritonavir	Sedation	6	373	22	66,506	48.63 ± 2.45
	Ritonavir	Liver injury	32	373	151	66,506	37.79 ± 1.45
	Tofacitinib	Red blood cell sedimentation rate increased	4	370	30	66,509	23.97 ± 2.83
	Tofacitinib	Blood cholesterol increased	13	370	125	66,509	18.69 ± 1.76
	Tofacitinib	Drug intolerance	5	370	52	66,509	17.28 ± 2.49
Monoclonal antibodies	Bamlanivimab	Nasal discomfort	10	4,923	15	61,956	8.39 ± 2.22
	Bamlanivimab	Troponin increased	35	4,923	62	61,956	7.10 ± 1.51
	Bamlanivimab	Body temperature increased	83	4,923	152	61,956	6.87 ± 1.31
	Bebtelovimab	Injection-related reaction	10	466	18	66,413	79.18 ± 2.16
	Bebtelovimab	Hyperventilation	6	466	22	66,413	38.87 ± 2.45
	Bebtelovimab	Fear	6	466	42	66,413	20.36 ± 2.34
	Casirivimab	Infusion-related reaction	89	415	2,130	66,464	6.69 ± 1.21
	Casirivimab	Blood pressure increased	25	415	621	66,464	6.45 ± 1.47
	Casirivimab	Hypoxia	39	415	985	66,464	6.34 ± 1.36
	Casirivimab\imdevimab	Infusion-related hypersensitivity reaction	33	3,414	7	63,465	87.64 ± 2.26
	Casirivimab\imdevimab	Seizure-like phenomena	9	3,414	11	63,465	15.21 ± 2.41
	Casirivimab\imdevimab	Pallor	70	3,414	107	63,465	12.16 ± 1.35
	Etesevimab	Pharyngeal paresthesia	4	1,141	19	65,738	12.13 ± 2.93
	Etesevimab	Flushing	131	1,141	651	65,738	11.59 ± 1.20
	Etesevimab	Flank pain	7	1,141	44	65,738	9.17 ± 2.22
	Imdevimab	Infusion-related reaction	88	395	2,131	66,484	6.95 ± 1.21
	Imdevimab	Hypoxia	40	395	984	66,484	6.84 ± 1.35
	Imdevimab	Blood pressure increased	24	395	622	66,484	6.49 ± 1.49
	Sarilumab	Jugular vein thrombosis	4	282	17	66,597	55.57 ± 2.96
	Sarilumab	Hepatocellular injury	27	282	119	66,597	53.58 ± 1.49
	Sarilumab	Atrioventricular block	5	282	36	66,597	32.8 ± 2.53
	Tocilizumab	Systemic infection	38	4,595	1	62,284	515.08 ± 7.28
	Tocilizumab	Hypofibrinogenemia	45	4,595	2	62,284	304.98 ± 4.12
	Tocilizumab	Pneumonia fungal	142	4,595	7	62,284	274.97 ± 2.14
Vaccines	azd-1222^b^	Vascular purpura	6	546	14	66,333	52.07 ± 2.59
	azd-1222	Peripheral artery thrombosis	5	546	18	66,333	33.75 ± 2.69
	azd-1222	Motor dysfunction	6	546	22	66,333	33.13 ± 2.46
	cx-024414^c^	Vascular purpura	5	495	15	66,384	44.70 ± 2.74
	cx-024414	Cardiomegaly	5	495	25	66,384	26.82 ± 2.60
	cx-024414	Malignant neoplasm progression	6	495	31	66,384	25.96 ± 2.39
	Tozinameran^d^	B-lymphocyte count decreased	24	3,271	8	63,608	58.34 ± 2.22
	Tozinameran	Vaccination failure	127	3,271	62	63,608	39.83 ± 1.35
	Tozinameran	Trigeminal neuralgia	13	3,271	8	63,608	31.60 ± 2.41

^a^ Unexpected ADEs marked in red.

^b^ azd-1222: Oxford/AstraZeneca COVID-19 Vaccine.

^c^ cx-024414: Moderna COVID-19 Vaccine.

^d^ Tozinameran: Pfizer–Biotech COVID-19 Vaccine.

**FIGURE 3:**
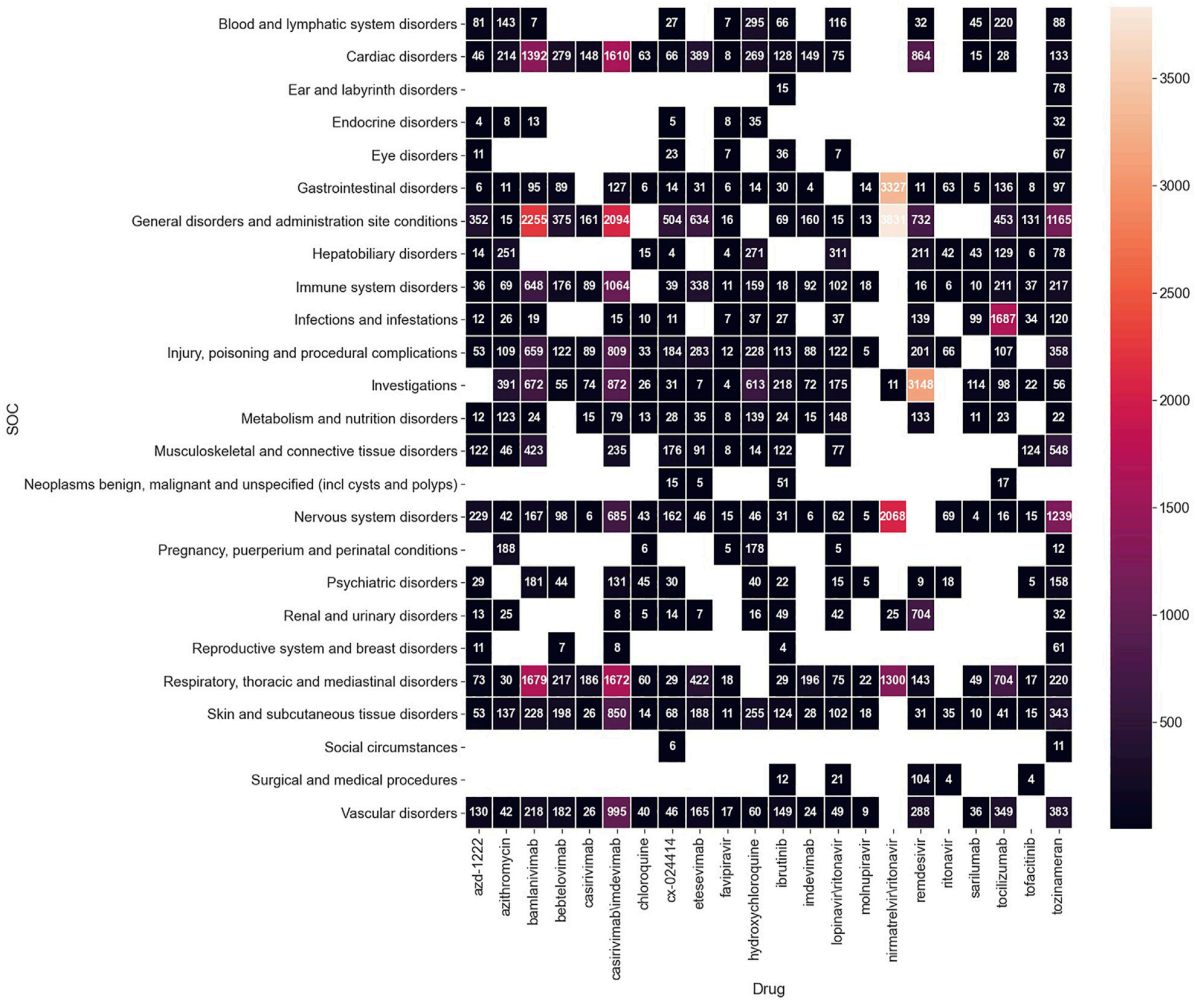
Number of reported cases of significant ADE signals on the SOC level.

After converting the signals from the PT to SOC level, 25 ADEs on the SOC level were recognized, and top signals included “general disorders and administration site conditions” (3,831 cases), “gastrointestinal disorders” (3,327 cases), and “nervous system disorders” (2,068 cases) for nirmatrelvir\ritonavir, and “investigations” (3,148 cases) for remdesivir. The numbers of reported cases of significant signals on the SOC level are shown in Figure 3.

**FIGURE 4:**
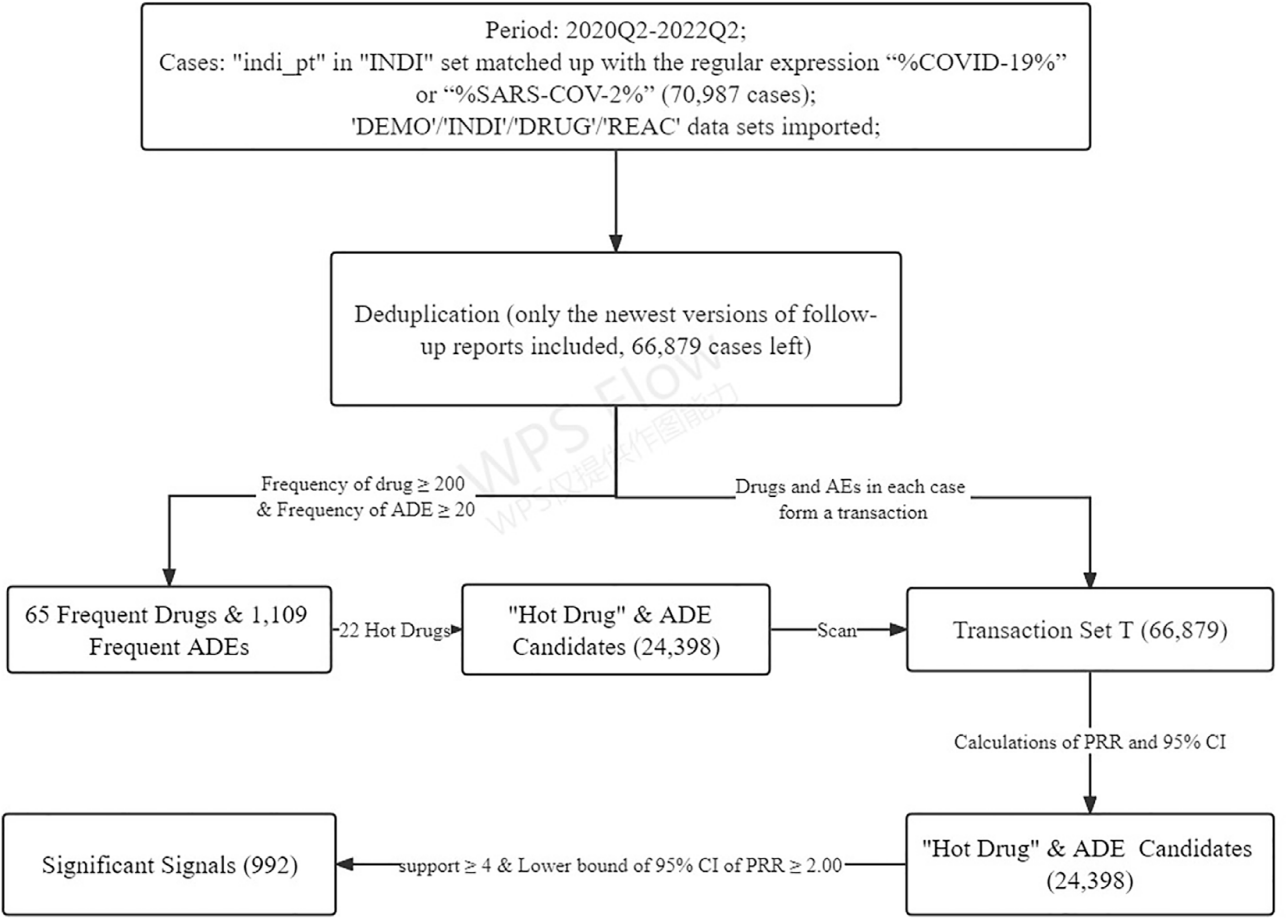
Flowchart of the data mining algorithm.

A flowchart was drawn to show the complete data mining process (Figure 4).

## 4 Discussions

From the 2nd quarter of 2020, over 70,000 ADE cases associated with COVID-19 have been reported to the FAERS, and our purpose was set to give an overview on the associations between some hot drugs and ADEs in the treatment and prevention of COVID-19 based on the data the FAERS had accumulated.

Although many of these ADE signals we observed may be regarded as possible complications of COVID-19 *per se*, those with relatively high PRRs and large reported cases (i.e., a and a + b) should still be paid attention to as they might give additional support or clues for further research.

In our study, a number of expected ADE signals on the PT level were observed such as “creatinine renal clearance decreased” (95% CI of PRR = 137.80 ± 4.17) and “liver function test increased” (95% CI of PRR = 29.84 ± 1.23) of remdesivir, “acute generalized exanthematous pustulosis” (95% CI of PRR = 46.53 ± 1.77) and “torsade de pointes” (95% CI of PRR = 33.68 ± 1.62) of hydroxychloroquine, and “ventricular arrhythmia” (95% CI of PRR = 30.27 ± 2.43) and “cardiovascular insufficiency” (95% CI of PRR = 22.07 ± 2.28) of azithromycin (Drugs.com, 2021). Some of these signals were also consistent to other data mining research studies conducted before (Gérard et al., 2021; Ngai et al., 2022). In addition, for azithromycin and remdesivir, we noticed that all the top 3 signals are associated with the heart and liver. Thus, when prescribing these medicines, we recommend physicians to be particularly careful about these ADE possibilities.

Among the unexpected signals, two strong ones (disease recurrence for nirmatrelvir\ritonavir and “hypofibrinogenemia” for tocilizumab) stood out, and we would make further discussions on them. For the other weaker signals [e.g., “methemoglobinaemia” (95% CI = 31.41 ± 2.20) for hydroxychloroquine and “eosinophilia” for lopinavir\ritonavir (95% CI = 49.62 ± 1.45)], limited by sparse clinical data, we assumed more research might be warranted.

In addition, we would also discuss some ADE signals on the SOC level for an easier understanding of the research result.

### 4.1 Signals on the PT level

#### 4.1.1 Disease recurrence of nirmatrelvir\ritonavir

Nirmatrelvir/ritonavir is an experimental combination protease inhibitor which blocks the replication of SARS-CoV-2, and the FDA has authorized the emergency use of this medication for the treatment of mild-to-moderate COVID-19 in adults and people over 12 years (weighing ≥40 kg) testing positive for COVID-19, and who are at high risk for progression to severe COVID-19, including hospitalization or death (Takashita and Yamayoshi, 2022).

There have been some case reports on “rebound” COVID-19 (recrudescent symptoms with a return of positive rapid antigen testing) after initial improvement and negative testing following the completion of the 5-day nirmatrelvir/ritonavir treatment (Ranganath et al., 2022).

The signals disease recurrence (a = 3,463, a + b = 9,517, 95% CI of PRR = 302.50 ± 1.27) and “symptom recurrence” (a = 227, a + b = 9,517, 95% CI of PRR = 136.82 ± 1.88) of nirmatrelvir/ritonavir in our study confirmed this possible ADE. We could also see that over 1/3 of the nirmatrelvir/ritonavir-associated FAERS cases ever reported this ADE, which may imply a relatively high frequency of symptom rebound after nirmatrelvir/ritonavir treatment.

#### 4.1.2 Hypofibrinogenemia of tocilizumab

The WHO recently made a strong recommendation to use IL-6 receptor blockers (tocilizumab and sarilumab) in patients with severe or critical COVID-19 (World Health Organization, 2021c).

Common ADEs of IL-6 receptor inhibitors include nasopharyngitis, headache, upper respiratory tract infection, gastritis, rash, arthralgia, extremity pain, fatigue, and nausea. Infections are the most frequent serious adverse events reported. Gastrointestinal perforation can occur in adults. Laboratory abnormalities include neutropenia, thrombocytopenia, dyslipidemia, and elevated liver enzymes (Campbell et al., 2011). In our study, a strong unexpected signal hypofibrinogenemia (95% CI of PRR = 304.98 ± 4.12) was observed for tocilizumab, which means in the cases where hypofibrinogenemia was reported, tocilizumab almost always got involved except only two cases.

Hypofibrinogenemia is defined as unusually low plasma concentrations of fibrinogen below the normal range of 2–4 g/L (especially ≤1.5 g/L). Human fibrinogen (i.e., coagulation factor I, FG, and FBG) is a 340 kD hexameric glycoprotein (GP) which is the precursor to fibrin and produced exclusively by the liver. During clotting, fibrinogen is converted to fibrin and the latter polymerizes and provides a major structure component of the clot. Acquired hypofibrinogenemia can be caused by liver disease, plasma exchange therapy, and consumptive coagulopathies such as DIC. Usually, as an acute phase reactant, fibrinogen could be elevated by 2- to 20-fold in an acute phase response (Green and Humphries, 1989; Rosenson, 1993). A decrease of fibrinogen is a strange phenomenon in COVID-19 patients. Only a few research studies ever reported this ADE of tocilizumab (but not in COVID-19 patients) (Martis et al., 2017; Üsküdar Cansu et al., 2019). Thus, we thought additional attention should be paid to this signal.

### 4.2 Signals on the SOC level

Figure 3 shows the number of cases for each signal on the SOC level to help readers, in combination with the Supplementary Appendix, get a whole picture of our data mining result. What has to be emphasized is that the interpretation of a signal should be based both on the number of cases and the frequency of the drug. We assumed that the larger these two parameters are, the more meaningful the signal would be. Some examples are given as the following.

“Cardiac disorders” was found strongly associated with casirivimab\imdevimab (1,610/3,414 cases), bamlanivimab (1,392/4,923 cases), and remdesivir (864/6,762 cases). According to *Drugs.com*, casirivimab\imdevimab and bamlanivimab are both experimental medicines in treating coronavirus. The data on the efficacy and safety of the two remains sparse, and their possible cardiac side effects may include chest pain and arrhythmias. We recommend physicians to be cautious about this ADE risk when prescribing these two medicines. Cardiac risk of remdesivir is a relatively well-confirmed ADE, and the signal of our research also stands behind it (Drugs.com, 2021; Attena et al., 2021).

“Infections and infestations” stood out for the monoclonal antibodies —tocilizumab (1,687/4,595 cases) and sarilumab (99/282 cases). This is consistent with the hypothesis that the use of IL-6 pathway inhibitors may be associated with an increased risk of secondary infections (Guaraldi et al., 2020; Busani et al., 2021).

“Investigations” is also an important SOC signal in Figure 3 with which 72 PTs (seen in the Supplementary Appendix) are associated in total. There are 3,148 cases reporting “investigations” for remdesivir, referring to the Supplementary Appendix, we could see that most of them are liver or kidney injury associated. This also supports the potential hepatoxicity and nephrotoxicity of remdesivir.

### 4.3 Limitations

Our study gave a brief on the potential ADE risk based on the COVID-19-associated cases of the FAERS in order to give some clues to ADE discovery and, if possible, help health professionals timely recognize ADEs and adjust their therapies. However, there are limitations in our study. First, data mining could only reflect some associations among “items” and does not provide enough evidence on causality. Second, as a spontaneous reporting system (SRS), the FAERS collected suspected ADE reports without requiring concrete evidence to prove the causalities between ADEs and drugs, and is also subject to reporting biases (i.e., reporters tend to report the ADEs which are of interest to them or maybe more confirmed by the public), which inevitably lead to under- or over-reporting. Third, there are significant differences in the frequencies of hot drugs (e.g., 9,517 cases for nirmatrelvir/ritonavir vs. 282 for sarilumab), and it is obvious that the lower the frequency, the less reliable the signal is. Fourth, the PRRs and their 95% CIs in this study were calculated against only the 60,000s COVID-19-associated cases, not the whole FAERS data; limited by the relatively small data amount, the possibility of false signals will increase. Last, for the ADEs of COVID-19 vaccines, the US FDA maintains another ADE reporting system (i.e., Vaccine Adverse Event Reporting System, VAERS), and the FAERS only received a relatively small number of the reports on vaccines. Thus, another ADE data mining work on COVID-19 vaccines based on the VAERS might be needed.

## 5 Conclusion

As one of the primary spontaneous reporting systems, the FAERS has accumulated a lot of ADE data which could be a great resource for data mining. COVID-19 is an emergent situation; many off-label therapies may bring too much ADE risk to the patients. Since the outbreak of COVID-19, the FAERS also has received quite a lot of ADE reports associated with COVID-19. Some data mining works also have been performed trying to figure out some meaningful signals. However, these works focused either only on some certain ADEs or only on some certain kinds of medicines (Ngai et al., 2022; Wu et al., 2022; Zhao et al., 2022). Hence, a work providing a whole picture based on the FAERS might be imperative and more helpful.

As far as we know, our work is the first, most comprehensive, and updated data mining based on the COVID-19 cases of the FAERS for recognitions of ADEs associated with COVID-19 therapies and prophylaxis. The result we offered in this article is a timely and convenient assistance and a panorama for pharmacovigilance in the containment of COVID-19, and may be helpful, if possible, for physicians to timely recognize any ADEs for their patients.

## Data Availability

The original contributions presented in the study are included in the article/Supplementary Material; further inquiries can be directed to the corresponding author.
